# CT and MR imaging of cystic renal lesions

**DOI:** 10.1186/s13244-019-0826-3

**Published:** 2020-01-03

**Authors:** Francesco Agnello, Domenico Albano, Giuseppe Micci, Giuseppe Di Buono, Antonino Agrusa, Giuseppe Salvaggio, Salvatore Pardo, Gianvincenzo Sparacia, Tommaso Vincenzo Bartolotta, Massimo Midiri, Roberto Lagalla, Massimo Galia

**Affiliations:** 1 Dipartimento di Biomedicina, Neuroscienze e Diagnostica Avanzata, Università degli studi di Palermo, Via del Vespro 127, 90127 Palermo, Italy; 2 Unità di Radiologia Diagnostica ed Interventistica, IRCCS Istituto Ortopedico Galeazzi, Via Riccardo Galeazzi 4, 20161 Milan, Italy; 3 Dipartimento di Discipline Chirurgiche Oncologiche e Stomatologiche, Università degli Studi di Palermo, Via Liborio Giuffrè 5, 90127 Palermo, Italy; 4 Dipartimento di Radiologia, Fondazione Istituto Giuseppe Giglio, Contrada Pietrapollastra, Via Picciotto, 90015 Cefalù (Palermo), Italy

**Keywords:** Bosniak, Cystic renal lesion, Cystic renal cell carcinoma, CT, MR

## Abstract

Cystic renal lesions are a common incidental finding on routinely imaging examinations. Although a benign simple cyst is usually easy to recognize, the same is not true for complex and multifocal cystic renal lesions, whose differential diagnosis includes both neoplastic and non-neoplastic conditions. In this review, we will show a series of cases in order to provide tips to identify benign cysts and differentiate them from malignant ones.

## Key points

- Cystic renal lesions are a common incidental finding on routinely imaging examinations.
- Benign simple cyst is usually easy to recognize at imaging.
- Differential diagnosis of complex and multifocal cystic renal lesions include both neoplastic and non-neoplastic conditions.
- The most widely used system to classify cystic renal lesions was introduced by Bosniak in 1984 and revised in 1997.
- Renal cysts can be divided into focal and multifocal.

## Introduction

Cystic renal lesions are very commonly encountered at abdominal ultrasound, computed tomography (CT), and magnetic resonance (MR) imaging. Most lesions are asymptomatic and incidentally found, but they can rarely manifest with abdominal pain, hematuria, and signs of infection (e.g., fever). Although the majority represents simple cysts, their pathologic spectrum is broad and includes developmental, neoplastic, and inflammatory processes.

Ultrasound represents usually the first-line imaging examination of the abdomen and kidney and can easily recognize simple, fluid-filled renal cysts with the following criteria: homogeneous anechoic content, marked posterior enhancement, and well-defined borders [1, 2].

When these criteria are absent, a cystic renal lesion is classified as a complex cyst [1, 2].

**Fig. 1:**
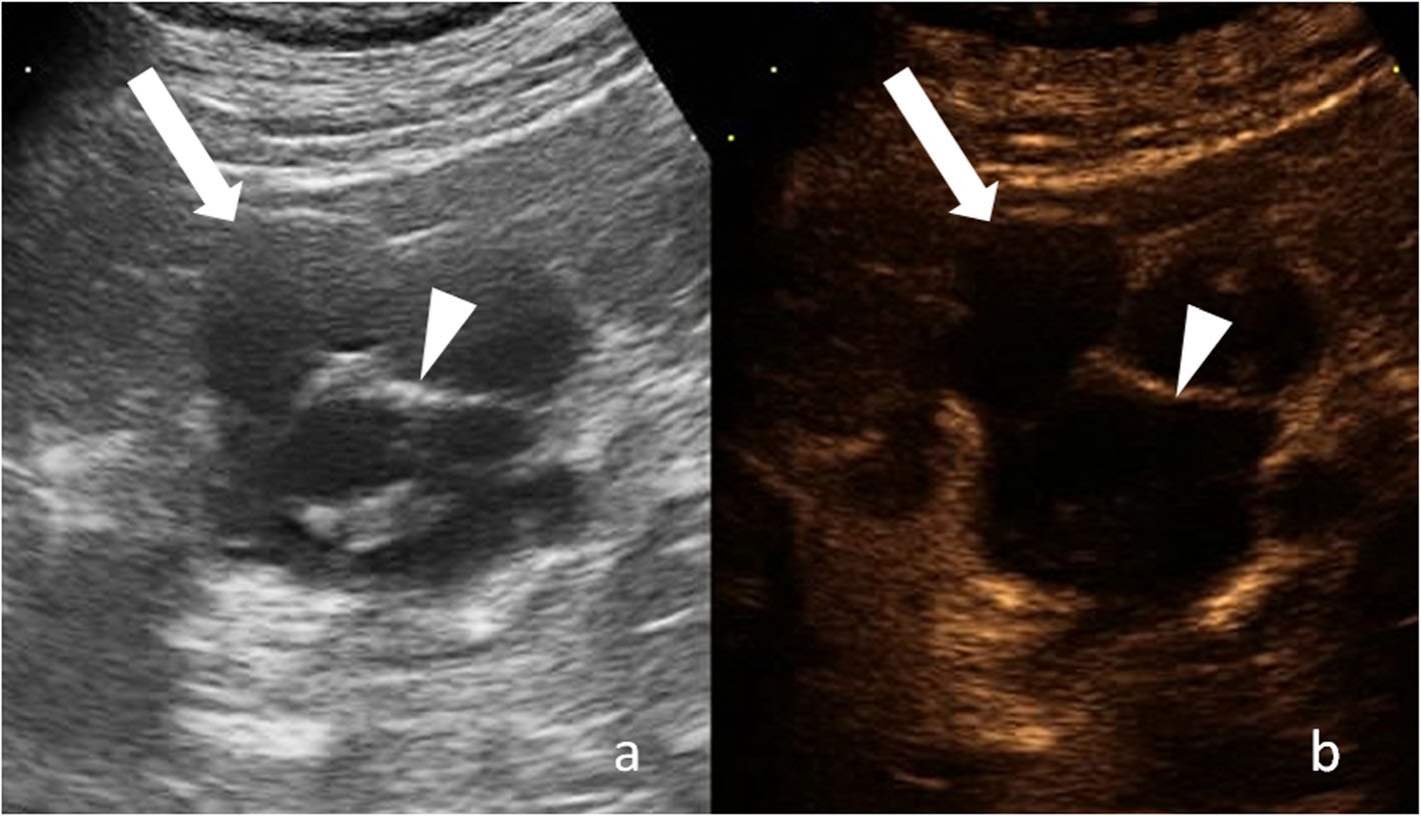
Cystic renal lesion in a 76-year-old-man. **a** Gray-scale ultrasound shows a cystic lesion (arrow) with a thin wall and thin septa (arrowhead), which contains fine calcifications. **b** Corresponding CEUS image shows enhancement of cyst wall and septa

The term “complicated cyst” must be reserved to those cysts, which undergo morphological changes due to documented rupture, hemorrhage, or infection [1, 2]. Complex and complicated renal cysts cannot be accurately characterized at ultrasound and usually warrant contrast-enhanced CT or magnetic resonance (MR) imaging [1, 2]. Because of absence of ionizing radiation and low-cost contrast-enhanced US (CEUS) is emerging as a valuable alternative to contrast-enhanced CT and MR [3, 4]. A growing body of evidence suggests that CEUS is useful to evaluate the vascularity of both cystic renal and hepatic lesion in real time using microbubble-based, purely intravascular, contrast agents (Fig. 1) [3–7]. However, the use of CEUS hampered operator dependency and technical limitations due to deep lesion location, bowel interposition, patient body habitus, and patient cooperation [3, 8]. Knowledge of the imaging characteristics and understanding the pathophysiology of cystic renal lesions helps the radiologist to derive the correct diagnosis.

A useful strategy for the evaluation of renal cysts is to divide them into focal and multifocal.

In this paper, we will expose radiologists to a series of CT and MRI cases in order to provide tips to identify benign cysts and differentiate them from malignant ones in adult patients.

## CT or MRI: advantages and disadvantages

Contrast-enhanced CT is the modality of choice in evaluating cystic renal masses. Narrow detector thickness (< 1 mm) and intravenous administration of contrast agent are mandatory to detect thin septa and small enhancing nodules [9]. Also, demonstration of enhancing areas helps differentiate solid components from hemorrhage or debris [10]. MRI is used when CT is contraindicated (e.g., patients with allergy to iodinated contrast agent) or as a problem-solving modality for equivocal findings. Indeed, MRI can show some septa that are less apparent at CT and demonstrate definitive enhancement in those cysts that show only equivocal enhancement at CT [11]. As a consequence, renal cysts can be placed in a higher Bosniak category with MRI than with CT [11].

## Focal renal cysts

Focal renal cysts are common in older subjects. Their prevalence, size, and number increase with age, with approximately 30% of people after the fourth decade and 40% after the fifth decade having at least one renal cyst [12, 13]. The majority is benign simple renal cysts and can be diagnosed with confidence. However, cystic renal lesions can have benign as well as malignant causes. Possible malignant causes include renal cell carcinoma (RCC) and metastasis. Since cystic RCCs, benign complicated cysts, and other cystic tumors can be radiologically indistinguishable, the goal of imaging when a renal cyst is found is to differentiate a benign “leave-alone” lesion from a lesion that requires treatment.

### Bosniak classification system for renal cysts

The most widely used system to classify cystic renal lesions was introduced by Bosniak in 1984 and revised in 1997 [14, 15]. This system was originally developed on CT findings, but it can be also used at MRI [11, 16, 17].

**Fig. 2:**
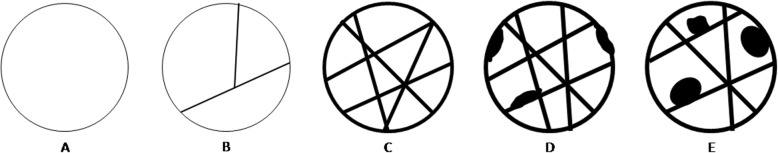
Imaging features of cystic renal lesions according to Bosniak classification. **a** Bosniak category I cyst: thin wall. **b** Bosniak category II cyst: thin wall; few, thin septa. **c** Bosniak category II-F cyst: minimally thickened wall; several, minimally thickened septa. **d** Bosniak category III cyst: irregularly thickened wall; several, irregularly thickened septa. **e** Bosniak category IV cyst: enhancing nodularity; irregularly thickened wall; several, irregularly thickened septa

Renal cysts are divided into five categories on the basis of imaging appearance (Table 1, Fig. 2). Each Bosniak category reflects the likelihood of cystic RCC that ranges from I (simple cyst) to IV (cystic tumors). Category I, II, and, IIF cysts are nonsurgical, while categories III and IV are surgical.

**Table 1:** The five categories of renal cysts, divided on the basis of imaging appearance

Bosniak category	Wall	Septa	Calcifications	Enhancing nodularity
I	Thin Non-enhancing	-	-	-
II	Thin Non-enhancing	Few Thin	Fine or slightly thickened	-
II-F^a^	Minimal thickening Perceived enhancement	Several Minimal thickening Perceived enhancement	Irregular or nodular	-
III^b^	Irregularly thick Measurable enhancement	Several Irregularly thick Measurable enhancement	Variable	-
IV	Irregularly thick Measurable enhancement	Several Minimal thickening Measurable enhancement	Variable	Wall and/or septa

^a^This category includes complicated (< 3 cm) cysts

^b^This category includes complicated (> 3 cm) cysts

Imaging findings include attenuation/signal intensity, size, presence of calcifications, septa and enhancing nodularity. Among these, enhancing nodularity is considered the most important predictor of malignancy [18]. At CT, enhancement requires an increase of attenuation of at least 15–20 HU from unenhanced to the contrast enhanced images [18]. A 10–15 HU change in attenuation can be due to incorrect placement of the region of interest, patient motion, or beam hardening from adjacent enhancing renal parenchyma (the so called “pseudoenhancement”) [19]. To overcome this problem, it has been suggested to use dual-energy CT, where true unenhanced images can be replaced by virtual unenhanced images [20]. Iodine quantification and iodine-related attenuation are used to differentiate nonenhancing cysts from enhancing solid masses [20].

In equivocal cases, another option is to use subtraction MRI to assess the presence or absence of enhancement [21].

Septa are defined as dividing wall within a renal cyst and are best appreciated at MRI than at CT. When present, they can be classified as thin, minimally thickened, or grossly thickened and irregular, and as enhancing or non-enhancing.

Calcifications are usually easy to recognize at CT but may be unapparent at MRI. Despite the importance in predicting the malignancy of solid renal masses, calcifications have limited utility in the Bosniak classification since they can be found in the wall or septa of both benign and malignant cysts [22]. Similarly, the size does not reliably predict the benignity or malignity of a renal cyst. Indeed, larger cysts can be benign and small ones can be malignant.

#### Category I renal cysts

**Fig. 3:**
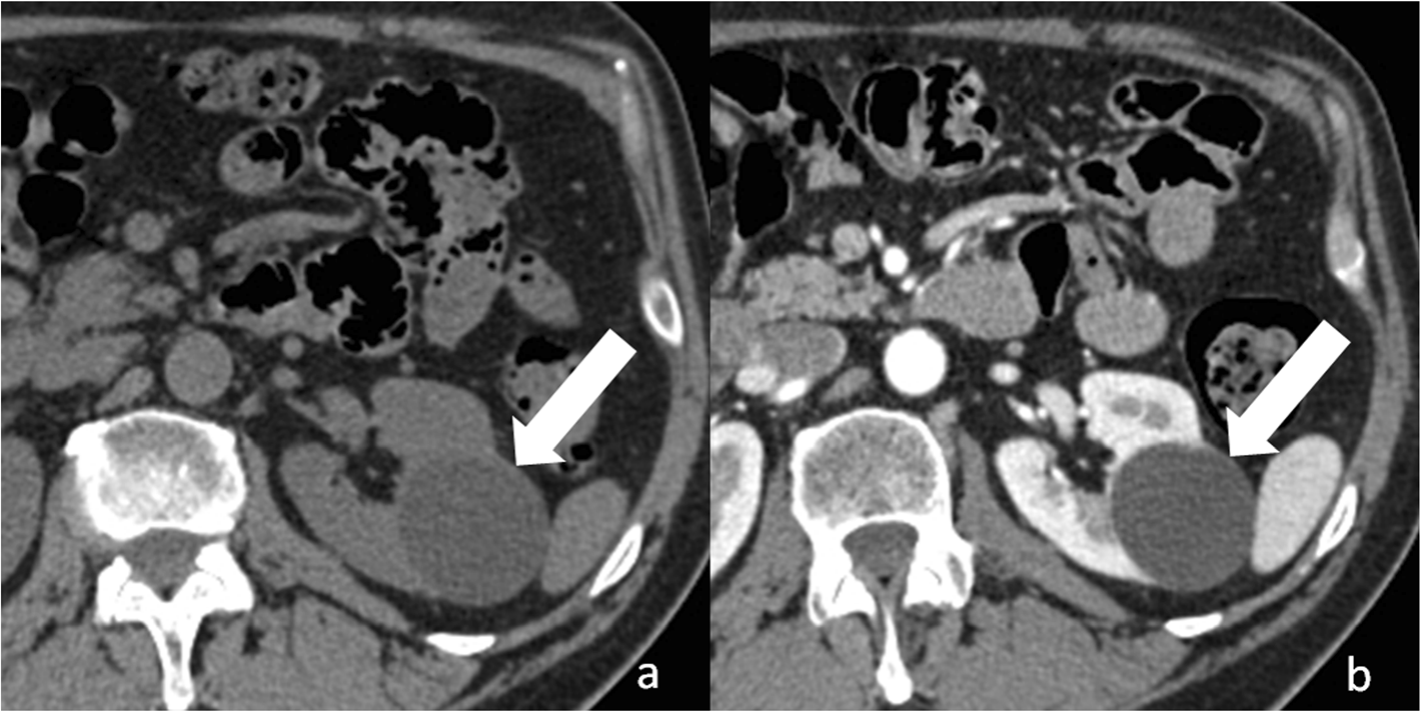
Bosniak category I renal cyst. Axial non-enhanced (**a**) and contrast-enhanced (**b**) CT images shows a cyst (arrow) with a thin and non-enhancing wall

**Fig. 4:**
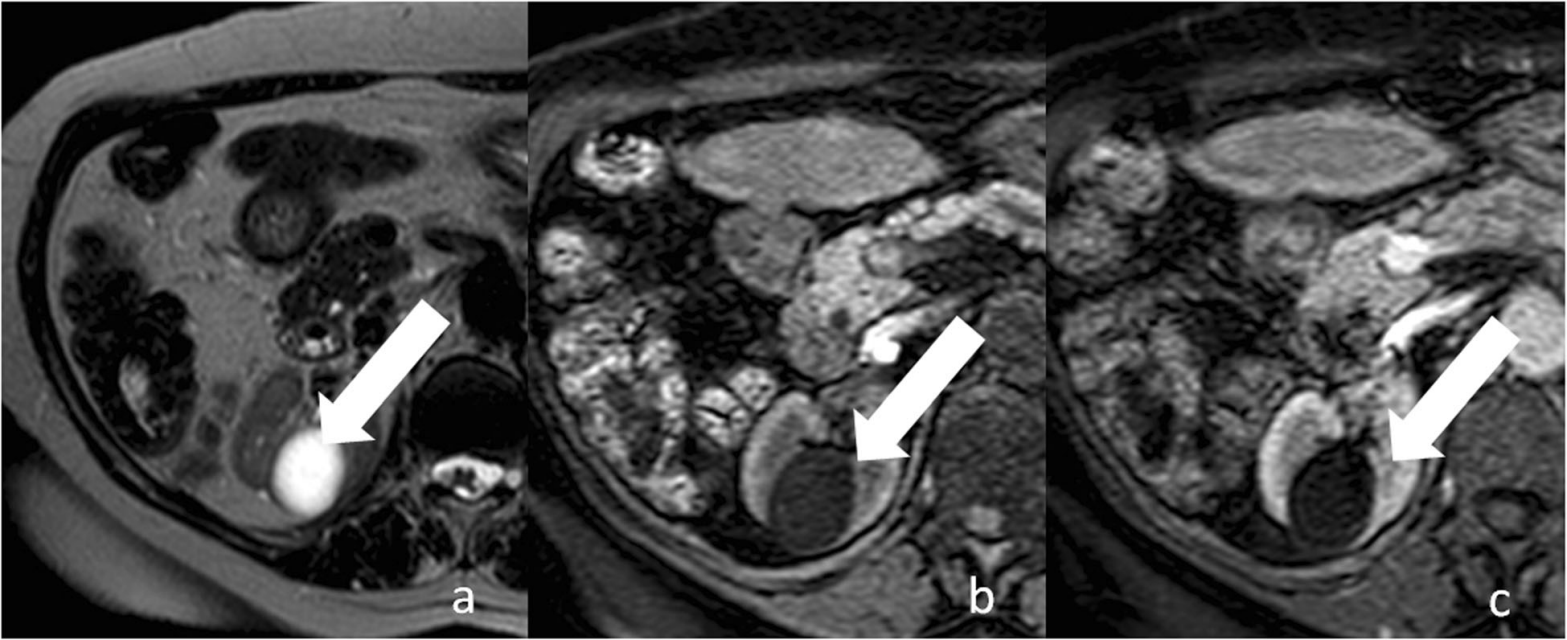
Bosniak category I renal cyst. **a** Axial T2-weighted MR image shows a lesion (arrow) with strong hyperintensity and a thin wall. Corresponding axial non-enhanced (**b**) and contrast-enhanced (**c**) T1-weighted MR images show a hypointense lesion with a thin and non-enhancing wall

Category I cysts are simple benign cysts. The exact pathogenesis is unknown. It has been suggested that they originate from weakening of the basement membrane of distal convoluted or collecting tubules [23]. Imaging appearance is consistent with water content: 0–20 HU attenuation on unenhanced CT, strong hyperintensity on T2-weighted MRI sequences, hypointensity on T1-weighted MRI sequences (Figs. 3 and 4). The wall is thin, hair-line, and non-enhancing. Calcifications, septa, and enhancing nodularity are absent. Almost all are benign. In a study including 1700 individuals with at least one renal cyst, only two patients developed a renal neoplasm [12]. Category I renal can grow in size over time. Treatment or follow-up are not recommended.

#### Category II renal cysts

**Fig. 5:**
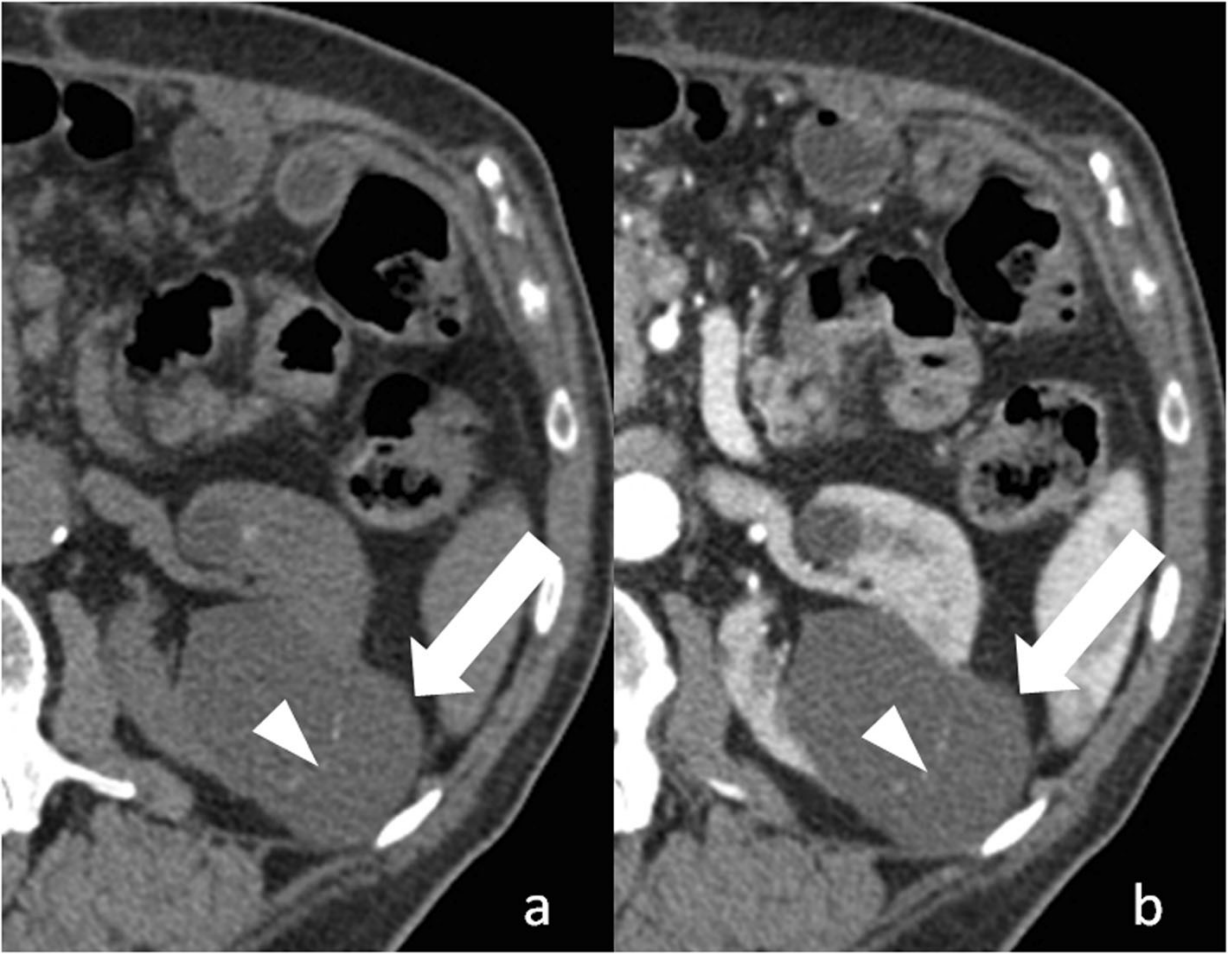
Bosniak category II renal cyst. **a** Axial non-enhanced CT image shows a lesion with a thin wall (arrow) and a thin septum (arrowhead), which contains fine calcifications. **b** Corresponding axial contrast-enhanced CT image shows enhancement of cyst wall and septum

Category II renal cysts are slightly more complicated in that they show hair-line wall, and few, thin septa, which can show perceived (not measurable) enhancement (Fig. 5). Fine calcifications or a short segment of slightly thickened calcifications can be present in the wall or septa. Complicated (proteinaceous or hemorrhagic) renal cysts measuring less than 3 cm are also included in the category II. These cysts show hyperattenuation (> 20 HU) on unenhanced CT, high signal intensity on unenhanced T1-weighted MRI sequences, and no enhancement, which helps differentiate benign cyst from RCC. Lesion homogeneity and smooth borders also are highly suggestive of a benign cyst [24]. In general, proteinaceous cysts measure 20–40 HU and are anechoic at ultrasound, while hemorrhagic cysts measure over 40 50 HU and can show a complex appearance at ultrasound [25]. Category II renal cysts are benign, and do not require treatment or follow-up.

#### Category IIF renal cysts

**Fig. 6:**
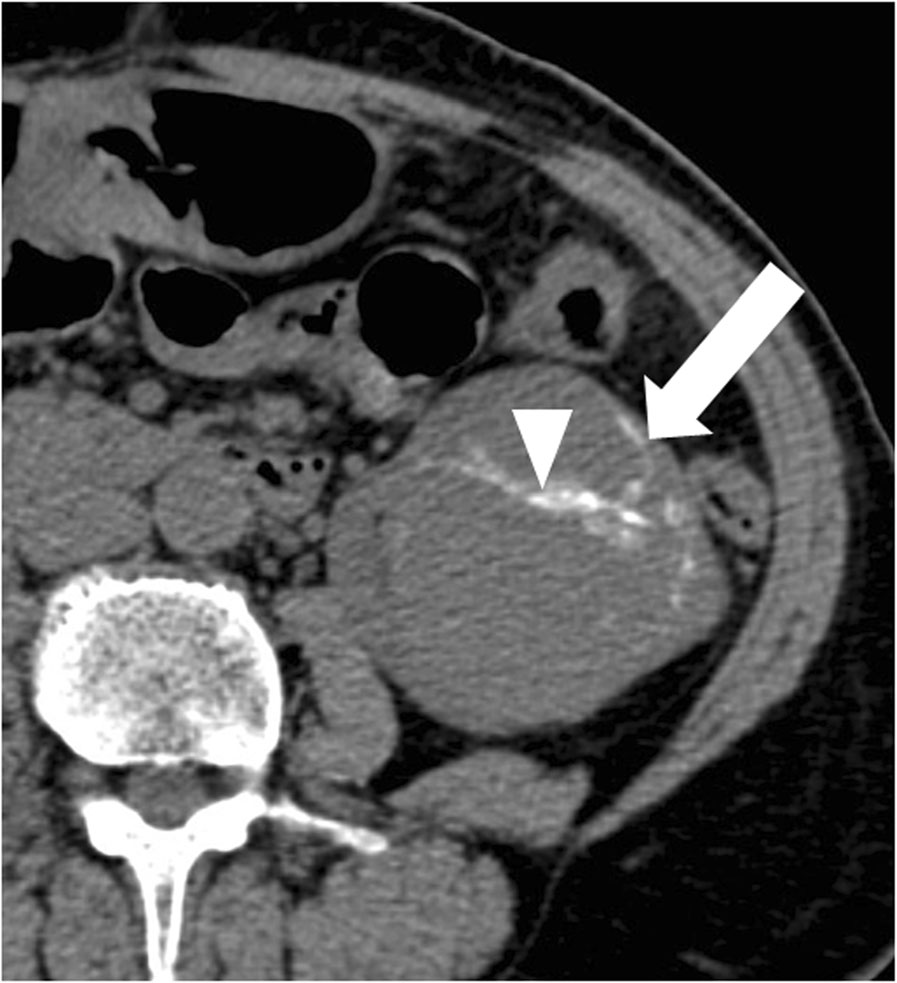
Bosniak category IIF renal cyst. Axial non-enhanced CT image shows a lesion with irregular calcifications within the wall (arrow) and septa (arrowhead)

**Fig. 7:**
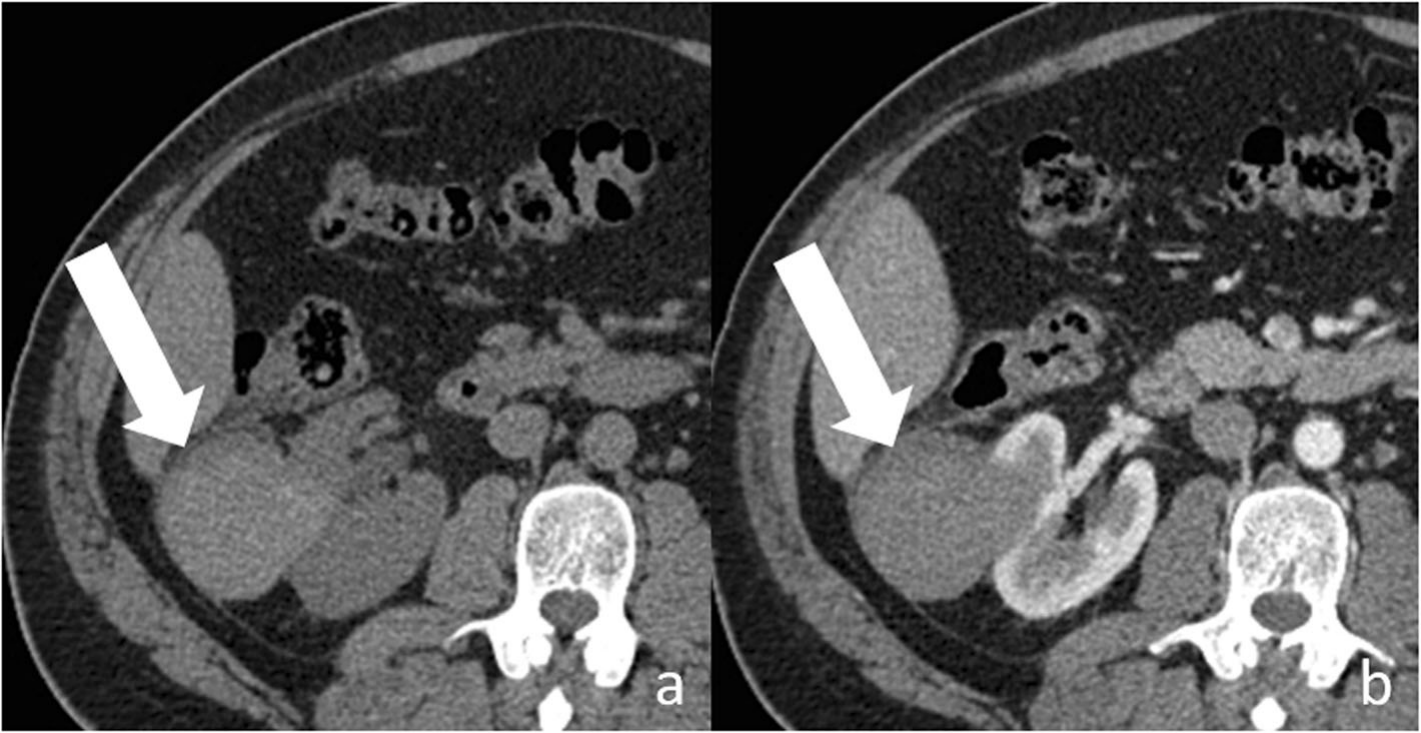
Bosniak category IIF renal cyst. Axial non-enhanced (**a**) and contrast-enhanced (**b**) CT images shows a large (> 3 cm) lesion (arrow) with spontaneous hyperattenuation and no enhancement

Category IIF renal cysts (“F” means follow-up) are more worrisome than category I and II [15, 26, 27]. The wall and septa can show minimal thickening and perceived (not measurable) enhancement and can contain irregular or nodular calcifications (Fig. 6). Unlike category II cysts, they can contain several septa. Complicated renal cysts measuring more than 3 cm are included in the category IIF (Fig. 7). Category IIF renal cysts are benign in 75–95% of time [28–30]. Imaging follow-up is required to exclude the malignancy by showing stability over time. However, the optimal interval time for follow-up is unclear and is influenced by cyst complexity. Bosniak had suggested that category IIF cysts with minimal complications need a 1–2-year follow-up, while more complex ones require at least a 3–4 year follow-up [31].

#### Category III renal cysts

**Fig. 8:**
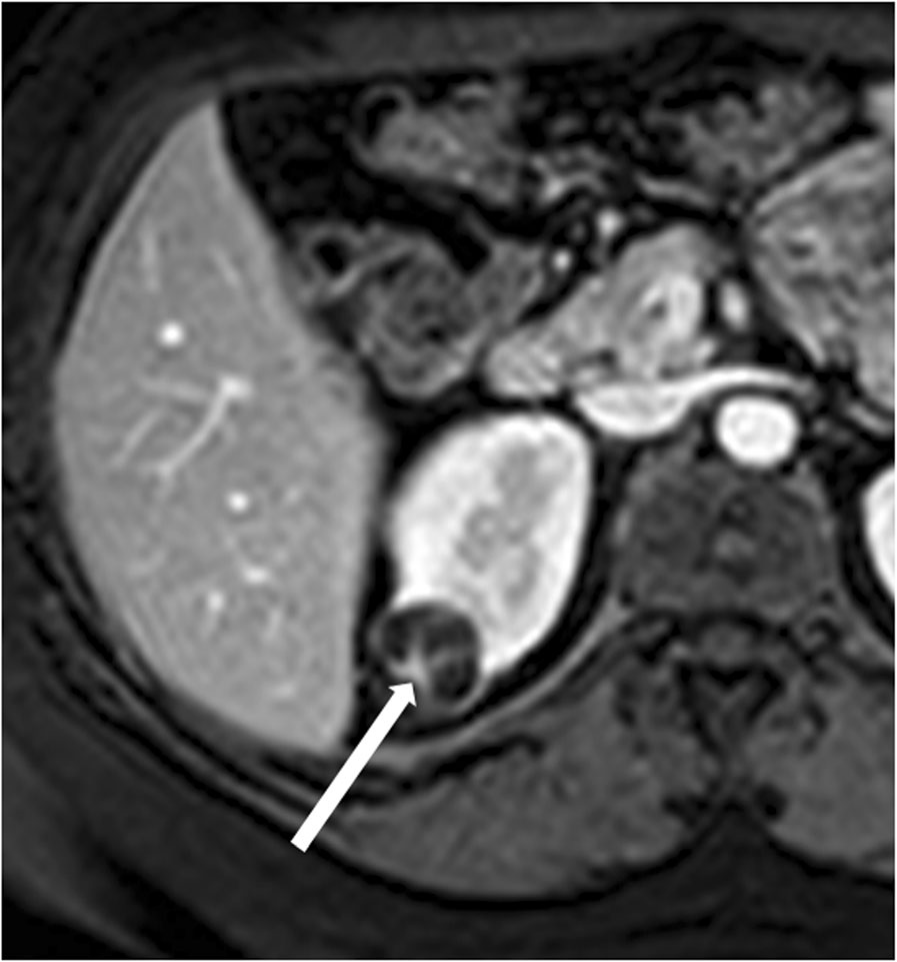
Bosniak category III renal cyst. Axial contrast-enhanced T1-weighted MR image shows a lesion with thick, enhancing wall and septa (arrow)

Category III renal cysts are indeterminate lesions with a reported malignancy of nearly 50% [28]. This category includes multilocular cysts, hemorrhagic and infected cysts, multilocular cystic nephroma, and cystic RCC [32]. Wall and septa are irregularly thick, show a measurable enhancement, and can contain thick nodular calcifications (Fig. 8). Septa are increased in number compared to category II cysts. Surgical removal of category III renal cysts is recommended because of their increased risk of malignancy.

#### Category IV renal cysts

**Fig. 9:**
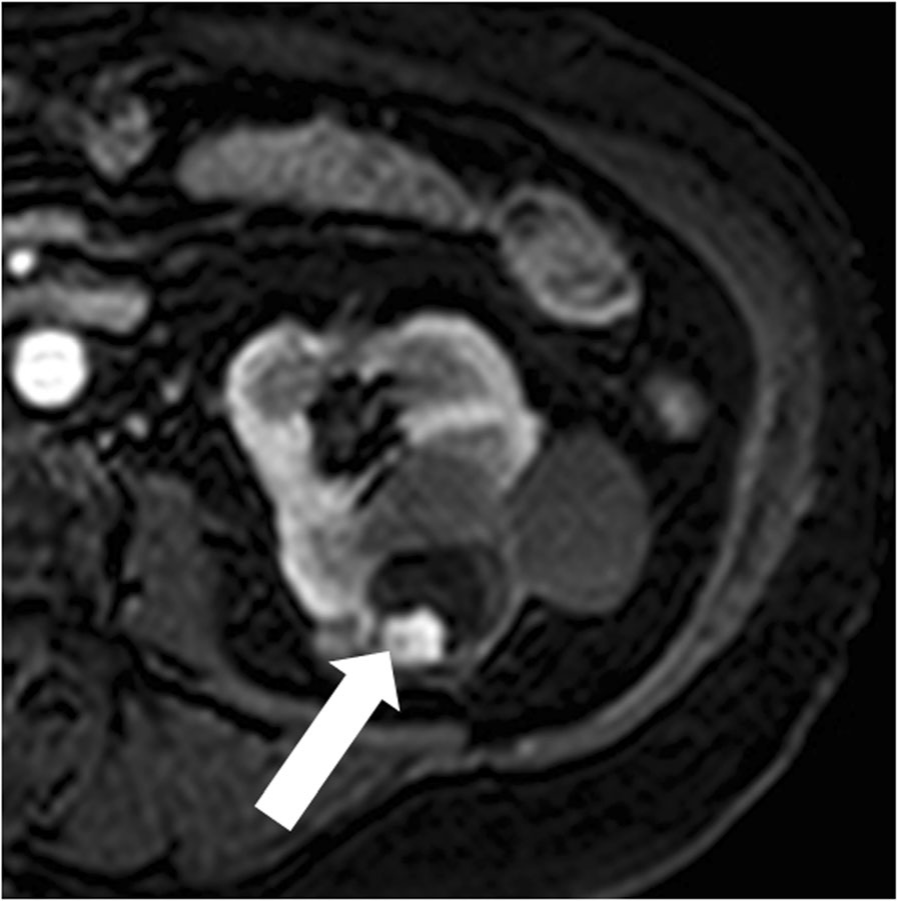
Bosniak category IV renal cyst. Axial contrast-enhanced T1-weighted MR image shows a lesion with a peripheral, enhancing, nodule (arrow)

Category IV renal cysts are considered malignant lesions. Nearly all are RCCs or, more rarely, metastases [32]. However, there are few benign lesions such as mixed epithelial and stromal tumor (MEST) and cystic angiomyolipomas that can be classified as category IV renal cysts [32]. The hallmark of this category is the presence of enhancing nodularity (Fig. 9). These cysts can also contain all findings observed in category III. Surgical removal is strongly recommended.

### Cystic renal cell carcinoma

Cystic RCC is relatively rare and comprises approximately 3–15 % of all cases of RCCs. It is found more commonly in younger age and in females compared with solid RCC [33]. The cystic appearance can be related to their inherent architecture or secondary to cystic degeneration and extensive necrosis [34]. Clear cell type RCC is the most common subtype, followed by papillary and chromophobe RCC. Clear cell type RCC can show a dominant cystic component or can arise in a simple cyst [35]. Multilocular cystic RCC of low malignant potential is a rare variant of clear cell type RCC with no reported recurrence or metastasis. This tumor is composed exclusively by cysts with low-grade tumor cell [36] and shows a variable imaging appearance, which ranges from category IIF to category IV renal cysts [35]. Papillary RCC can appear as a cyst with hemorrhagic or necrotic content and a thick pseudocapsule [35]. Cystic renal RCCs have a more favorable prognosis of all subtypes of RCC: they have a low Fuhrman grade, grow slowly, and rarely metastasize or recur [37].

### Renal metastases

Renal metastases are not uncommon, with reported frequencies ranging from 7 to 20% at post-mortem studies [38–41]. The most common primary malignancies are the lung, breast, gastrointestinal tract, and melanoma. CT and MR imaging diagnosis is less frequent because post-mortem studies included microscopic lesions, which are beyond CT resolution [42, 43].

Renal metastases can show a solid or cystic appearance. The differentiation of renal metastasis from RCC on the basis of CT and MR findings alone may be impossible [42–44]. However, some features are likely to be distinctive: renal metastases are frequently multiple, bilateral and small [42, 43].

### Mixed epithelial and stromal tumors

The MESTs area heterogeneous group of rare renal tumors occurring predominantly in perimenopausal women (female-to-male ratio, 11:1). The MEST appears as a well-marginated lesion with a variable proportion of solid and cystic components [45]. Septa and nodules can show heterogeneous and delayed enhancement [45]. MEST can show an exophytic growth or herniate into the renal pelvis [45]. Adult cystic nephroma is now classified within MEST family due to similar histologic and epidemiologic findings [36]. This tumor appears as an encapsulated lesion, with cysts of variable size, and thin, variably enhancing, septa [46]. Calcifications are peripheral and curvilinear [46]. Solid components are typically absent [46]. At MRI, the capsule and septa can show hypointensity on both T1- and T2-weighted images due to the fibrous composition. Since imaging features are non-specific, differentiation between MEST and cystic RCC requires pathologic examination.

### Renal abscess

**Fig. 10:**
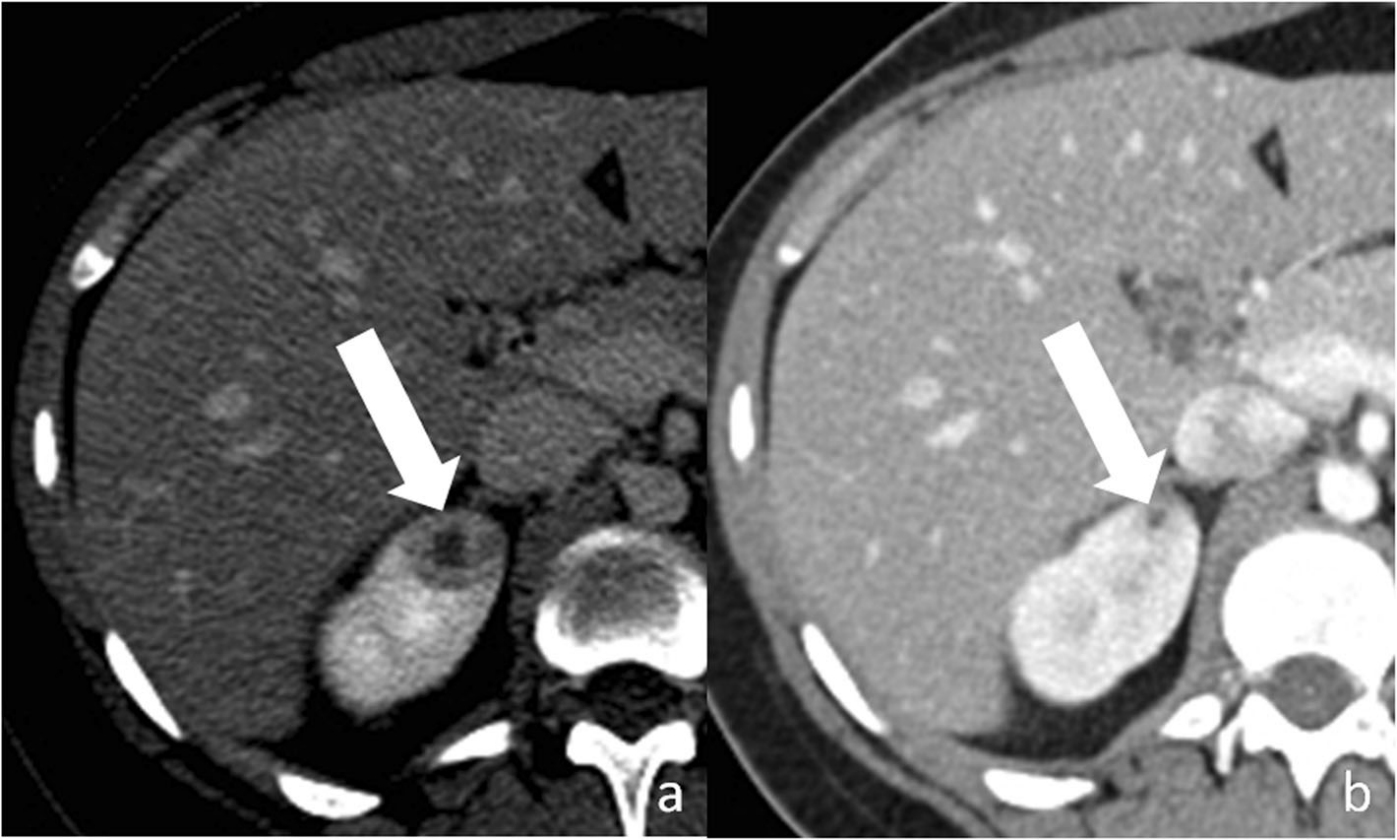
Renal abscess. **a** Axial contrast-enhanced CT shows a cystic lesion (arrow) with a peripheral, thick, enhancing, wall. **b** Axial contrast-enhanced CT obtained 3 months after antibiotic therapy shows decrease in size of the lesion

Renal abscess is an uncommon entity that usually results from a complication of untreated or inadequately treated acute pyelonephritis or ascending urinary tract infection. More rarely, it results from hematogenous spread from an extra-urinary source of infection (e.g., diverticulitis, pancreatitis). Patients may present with signs and symptoms of infection. Renal abscess can appear as a complex renal cyst with inhomogeneous areas of fluid attenuation/intensity and a thick and irregular wall that shows a little enhancement on excretory phase (Fig. 10). Because of the presence of viscous pus, the fluid component shows a characteristic strong and heterogeneous diffusion restriction on diffusion-weighted imaging, which favors the diagnosis of renal abscess over that of RCC [47]. Renal parenchyma around the abscess can show low density/intensity on early phases and delayed enhancement [48, 49]. Fat stranding is often found adjacent to the renal abscess [50]. Gas can be rarely present within the lesion and strongly suggests abscess formation. When imaging findings, clinical history and laboratory tests do not permit a confident differentiation between renal abscess and cystic RCC; biopsy/drainage should be performed to obtain the correct diagnosis.

## Multifocal renal cysts

Multifocal cystic renal diseases comprise a heterogeneous spectrum of hereditary and nonhereditary diseases characterized by the presence of multiple simple kidney cysts [32]. Hereditary entities are due to mutations of genes involved in the formation and functioning of renal cilia, which result in epithelial proliferation and development of renal cysts [51]. Autosomal dominant polycystic kidney disease is the most common hereditary multifocal renal disease*.* Nonhereditary entities are due to obstructive, stromal-epithelial malinductive and neoplastic mechanisms [52]. Most common causes of nonhereditary multifocal cysts formation include lithium-induced nephrotoxicity, acquired cystic renal disease, and localized cystic renal disease. The location and appearance of renal cysts, presence of interposed normal renal parenchyma, size of the kidneys, patient’s age at presentation, and degree of renal function help differentiate at imaging multifocal cystic renal diseases.

### Autosomal dominant polycystic kidney disease

**Fig. 11:**
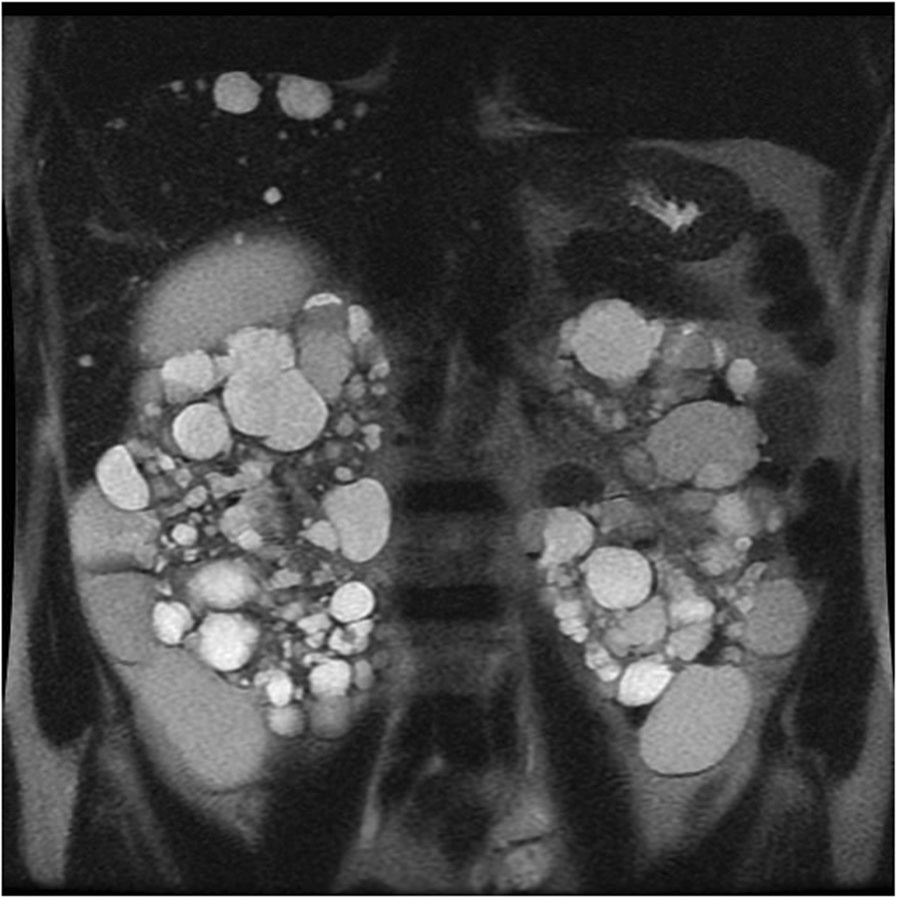
Autosomal dominant polycystic kidney disease. Coronal T2-weighted image shows symmetric enlargement of the kidneys, which contain multiple cysts with variable size

Autosomal dominant polycystic kidney disease (ADPKD) is the most common hereditary renal disorder and occurs in approximately one of 500 live births [51]. Mutations in one of the two genes encoding plasma membrane—spanning polycystin 1 and polycystin 2 (PKD1 and PKD2)—are responsible of the disease. It is characterized by progressive development and growth in size of simple renal cysts, leading to symmetric enlargement of the kidneys and chronic renal failure by late middle-age [52, 53] (Fig. 11). Cysts have variable dimension (from few millimeters to several centimeters) and are diffusely distributed through the kidneys. Cyst complications include hemorrhage, pyogenic infection, and, more rarely, rupture. The risk for RCC is not increased in comparison with the general population except in patients on dialysis [54]. The added risk of malignancy in dialysis patients is probably related to the effects of coexistent acquired cystic renal disease [54].

Hepatic cysts are the most common extra-renal manifestations of ADPKD and show variable number, size, location, and distribution [51, 54]. Polycystic liver disease is uncommon and leads to hepatomegaly [51, 54]. More rare hepatic complications include congenital hepatic fibrosis and segmental dilatation of biliary tract [54].

The other extra-renal manifestations of ADPKD include cysts in other organs such as pancreas and non-cystic abnormalities such as cardiac valvulopathies and intracranial aneurysms [51]. Imaging plays a crucial role in the identification of ADPKD in high-risk individuals (those with a positive family history). The diagnosis of ADPKD requires at least three renal cysts (unilateral or bilateral) in high-risk patients 15–39 years of age, at least two cysts in each kidney in high-risk patients 40–59 years of age, and several bilateral renal cysts in high-risk patients 60 years of age or older [55]. Since renal enlargement correlates with a decline of renal function, estimation of renal volume can predict the risk for renal failure [53].

### Acquired cystic renal disease

**Fig. 12:**
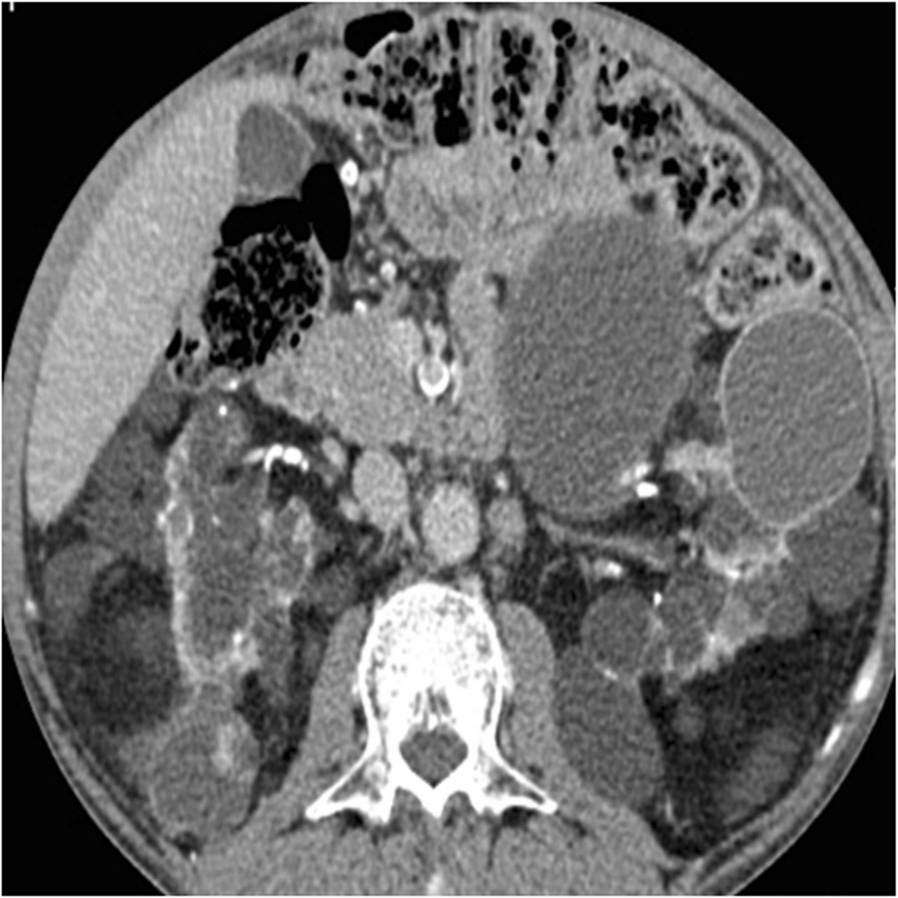
Acquired cystic renal disease. Axial contrast-enhanced CT image shows atrophic kidneys, which contain multiple cysts of variable size

Acquired cystic kidney disease (ACKD) is a consequence of sustained uremia in patient with end-stage renal disease [52]. The disease is found in 8–13% patients with end-stage renal disease and in approximately 50% patients on dialysis. The disease is multifactorial. It is the progressive destruction of renal functioning nephrons with compensatory hypertrophy of remaining renal parenchyma, obstruction of renal tubules by interstitial fibrosis or oxalate crystals, and cyst formation [52]. Kidneys are atrophic and contain multiple cysts with variable size (from few millimeters to several centimeters) and imaging appearance (Fig. 12). Since renal cysts are extremely common in the adult population, the diagnosis of ACKD requires the presence of three or more cysts in each kidney, in conjunction to end-stage renal disease, and no history of hereditable renal disease [56]. Cyst hemorrhage is a common complication and can cause hematuria, whereas cyst rupture, perinephric hematoma, and retroperitoneal hemorrhage are less frequent [52]. Development of RCC in the wall of the cyst is the most serious complication of ACKD, with a higher rate in comparison to the general population [55].

The most common tumor type in patients with ACKD is acquired cystic disease **(**ACD)-associated RCC, followed by papillary and clear cell type RCC [36]. ACD-associated RCC has unique morphologic features and is found exclusively in patients with end-stage renal disease and ACKD [36].

### Lithium-induced nephropathy

Long-term lithium therapy is a well-known cause of nephrotoxicity in the form of polyuria-polydipsia syndrome (diabetes insipidus) and chronic renal insufficiency [57]. Characteristic imaging findings include normal or slightly decreased size of kidneys with multiple, uniformly, and symmetrically distributed microcysts [58]. Microcysts measure 1–2 mm in diameter and are located in both cortex and medulla [58].

### Localized cystic renal disease

**Fig. 13:**
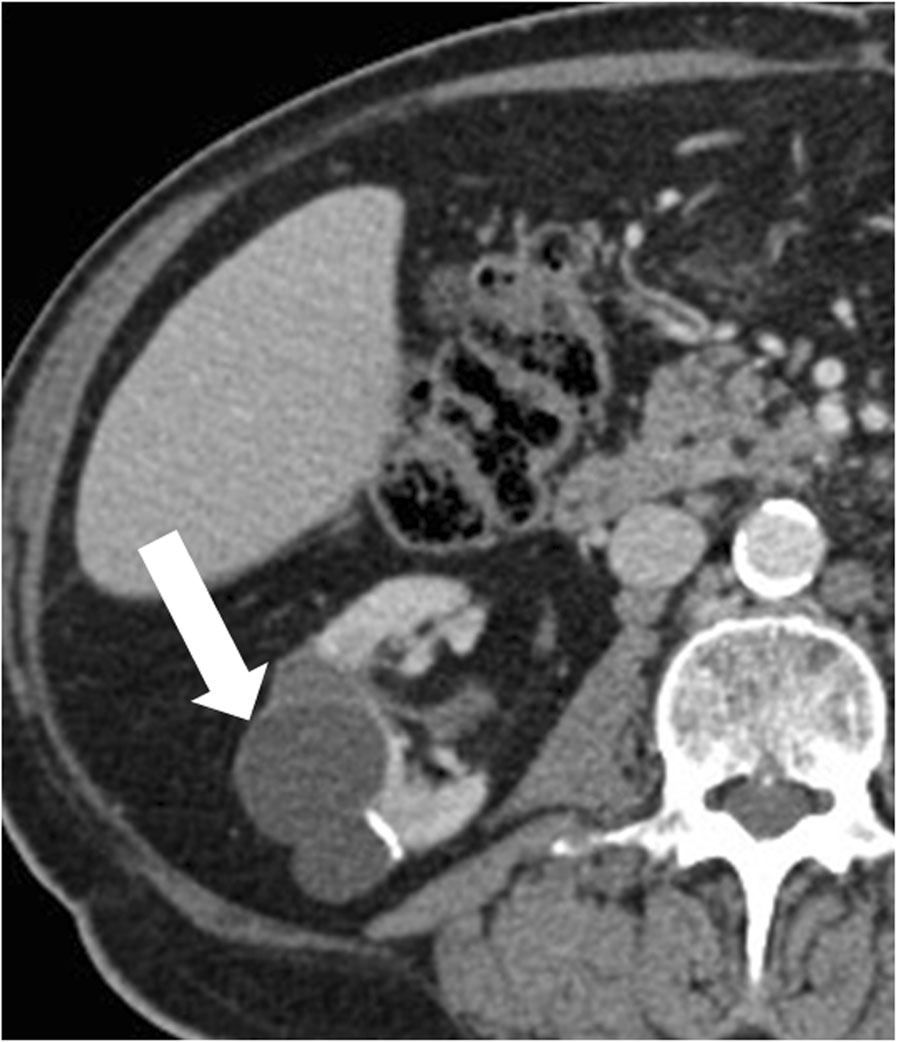
Localized cystic renal disease. Axial contrast-enhanced CT image shows a conglomeration of multiple simple cysts of variable size (arrow) in the right kidney

Localized cystic renal disease is a rare, nonhereditary, form of cystic renal disease, which manifests as a conglomeration of multiple simple cysts of variable size [59] (Fig. 13). In contrast to ACKD and ADPKD, localized cystic renal disease is typically unilateral and not progressive. The disease usually involves only a portion of the kidney with a polar predilection [59]. Entire renal involvement is rare [58]. The contralateral kidney is normal. The presence of interposed normal renal parenchyma and the absence of a capsule help to differentiate localized cystic renal disease from cystic nephroma and multiloculated cystic RCC [58]. Cystic involvement of other organs is typically absent [58].

## Conclusions

Cystic renal lesions are commonly encountered on radiologic examinations. Complex and multifocal cystic renal lesions are often a diagnostic challenge, since they can represent neoplastic and non-neoplastic conditions. The Bosniak classification system is a well-established imaging method, which helps radiologists and surgeons in daily practice in the differentiation of nonsurgical from surgical lesions. Radiologists should also recognize the imaging appearances of specific types of cystic lesions in order to better characterize them.

## Data Availability

Not applicable

## Abbreviations

ACKD: Acquired cystic kidney disease
ADPKD: Autosomal dominant polycystic kidney disease
CEUS: Contrast-enhanced US
CT: Computed tomography
MESTs: Mixed epithelial and stromal tumors
MRI: Magnetic resonance imaging
RCC: Renal cell carcinoma
